# Genome wide array-CGH and qPCR analysis for the identification of genome defects in Williams’ syndrome patients in Saudi Arabia

**DOI:** 10.1186/s13039-016-0266-4

**Published:** 2016-08-12

**Authors:** I. R. Hussein, A. Magbooli, E. Huwait, A. Chaudhary, R. Bader, M. Gari, F. Ashgan, M. Alquaiti, A. Abuzenadah, M. AlQahtani

**Affiliations:** 1 Centre of Excellence in Genomic Medicine Research (CEGMR), King Abdulaziz University, Jeddah, 21589 KSA Saudi Arabia; 2 Diagnostic Genomic Medicine Unit (DGMU), King Abdulaziz University, Jeddah, KSA Saudi Arabia; 3 Faculty of Science, King Abdulaziz University, Jeddah, KSA Saudi Arabia; 4 Faculty of Medical Sciences, King Abdulaziz University, Jeddah, KSA Saudi Arabia; 5 Pediatric Cardiology Department, King Abdulaziz University, Jeddah, KSA Saudi Arabia

**Keywords:** Williams Syndrome, Array-CGH, FISH, qPCR, Congenital heart defects

## Abstract

**Background:**

Williams-Beuren Syndrome (WBS) is a rare neurodevelopmental disorder characterized by dysmorphic features, cardiovascular defects, cognitive deficits and developmental delay. WBS is caused by a segmental aneuploidy of chromosome 7 due to heterozygous deletion of contiguous genes at the long arm of chromosome 7q11.23. We aimed to apply array-CGH technique for the detection of copy number variants in suspected WBS patients and to determine the size of the deleted segment at chromosome 7q11.23 in correlation with the phenotype. The study included 24 patients referred to the CEGMR with the provisional diagnosis of WBS and 8 parents. The patients were subjected to conventional Cytogenetic (G-banding) analysis, Molecular Cytogenetic (Fluorescent In-Situ Hybridization), array-based Comparative Genomic Hybridization (array-CGH) and quantitative Real time PCR (qPCR) Techniques.

**Results:**

No deletions were detected by Karyotyping, however, one patient showed unbalanced translocation between chromosome 18 and 19, the karyotype was 45,XX, der(19) t(18;19)(q11.1;p13.3)-18. FISH technique could detect microdeletion in chromosome 7q11.23 in 10/24 patients. Array-CGH and qPCR confirmed the deletion in all samples, and could detect duplication of 7q11.23 in three patients and two parents. Furthermore, the size of the deletion could be detected accurately by both array-CGH and qPCR techniques. Three patients not showing the 7q11.23 deletion were diagnosed by array-CGH to have deletion in chr9p13.1-p11.2, chr18p11.32-p11.21 and chr1p36.13.

**Conclusion:**

Both FISH and array-CGH are reliable methods for the diagnosis of WBS; however, array-CGH has the advantage of detection of genome deletions/ duplications that cannot otherwise be detected by conventional cytogenetic techniques. Array-CGH and qPCR are useful for detection of deletion sizes and prediction of the interrupted genes and their impact on the disease phenotype. Further investigations are needed for studying the impact of deletion sizes and function of the deleted genes on chromosome 7q11.23.

**Trial registration:**

ISRCTN ISRCTN73824458. MOCY-D-16-00041R1. Registered 28 September 2014. Retrospectively registered.

## Background

Congenital heart defects (CHDs) are major pediatric health problem in Saudi Arabia (SA). A prevalence of CHDs ranging between 2.1 and ~10.7/1000 live births was reported [1, 2], with the highest prevalence found in the Southwestern and the Northern Provinces [3]. A community-based National prevalence study of symptomatic CHDs reported a prevalence of 2.1/1000 children [4]. CHDs associated with other malformations present a challenging problem in clinical diagnosis and genetic counseling. Williams’s syndrome (WS) or Williams-Beuren syndrome (WBS) (OMIM 194050) is a contiguous gene syndrome caused by hemizygous deletion of chromosome 7q11.23 and is often associated with a CHD. Affected children have distinctive facial features, congenital heart defects mainly supra valvular aortic stenosis, cognitive deficits, unique personality characteristics and infantile hypercalcemia [5, 6]. Other features included stellate iris pattern, elfin facial features, coordination problems, developmental delay, short stature, and friendly personality [7, 8]. The disease occurrence is mostly sporadic with an estimated prevalence ranging between 1/7500 and 1/25,000 [9, 10].

Most cases of WBS occur as random event during formation of reproductive cells (eggs or sperm) in a parent of an affected individual. In a small percentage of cases patients inherit the chromosomal deletion from a parent with the condition [9, 11].

Accurate diagnosis is essential for providing optimal medical care and proper genetic counselling and estimation the risk of recurrence in future pregnancies. The disease is not easily detectable using conventional cytogenetic analysis due to limited resolution of (<5 Mb) and is usually detected by Fluorescent in-Situ Hybridization (FISH). The development of more recent techniques such as quantitative Real-Time Polymerase Chain Reaction (qPCR), and microarray-based Comparative Genomic Hybridization (array-CGH) has allowed for more accurate diagnosis [11].

Common deletion size causing WBS spans 1.5 to 1.8 million base pair (Mb) which contains more than 25 genes and it is believed that loss of several of these genes probably contributes to the characteristic features of this disorder [12]. The deleted region is flanked by three large low copy-repeat sequences (LCR) (320 Kb) known as LCR blocks A, B, and C which share high similarity of nucleotide sequences leading to non-allelic homologous recombination (NAHR) conferring liability to mispairing and unequal crossing over leading to deletions and duplications [13]. Smaller or larger deletions have been reported and a phenotypic variability was identified to correlate with the size of deletion in the WBS region [14]. The use of qPCR has allowed the precise definition of the deletion size and more accurate identification of genotype /phenotype correlation in patients with WBS [15, 16]. More recently, array-CGH technique has been applied for the accurate diagnosis of the deleted genes causing the typical and atypical phenotypes in WBS [17, 18].

Patients with WBS are not easily diagnosed early in neonatal life until characteristic personality and phenotypic features become apparent later in childhood. Furthermore, the phenotype becomes variable with advancing age which imposes more challenge for clinical diagnosis. To our knowledge no previous studies for the molecular diagnosis of WBS have been reported in Saudi Arabia. Therefore, we aimed to investigate the clinical utility of array-CGH technique for the early diagnosis of WBS, and investigate the impact of size of deletion on the phenotype.

### Subjects & methods

Twenty four patients suspected for WBS and 8 parents were referred from the Pediatric Cardiology Clinic, King Abdulaziz University Hospital (KAUH), to the Center of Excellence in Genomic Medicine Research (CEGMR) for genetic diagnosis. They were 11 M: 13 F, their ages ranged between 1 and 18 years old (mean = 5.52). Besides, samples from 20 normal subjects were used as controls. Peripheral blood samples were obtained on Sodium heparin for chromosomal and FISH analysis, and another sample was taken on EDTA for DNA extraction. All samples were subjected to conventional cytogenetic analysis using GTG-banding and (FISH) technique, as well as array-CGH and real time qPCR techniques. Peripheral blood samples were obtained from patients after taking informed consent from the patients or their parents or patient’s guardian. This study was approved by the Institutional Review Board and Ethical Committee of the (CEGMR), as part of a project funded by the KACST, King Abdulaziz University (KAU) (Code # 016-CEGMR-ETH).

## Methods

Conventional GTG-banding technique was applied for the chromosomal analysis of all patients as a routine procedure in the DGMU lab.

### Fluorescent In situ hybridization (FISH)

We used Vysis LSI ELN Probe, which is 180 kb Spectrum Orange directly labeled fluorescent DNA probe specific for the William’s region locus (7q11.23-) and LSI D7s probe which is 108 kb Spectrum Green directly labeled fluorescent DNA probe specific for the region located in the (7q31). The probe tests the presence or absence of ELN and LIMK1 genes on chromosome 7q, control loci D7S613 genes were involved in “William’s region” for validation of the reaction. All steps of hybridization and washing procedure were done following the manufacturer instructions (Vysis).

### Array-CGH technique

We applied the high resolution oligonucleotide-based 2x400 array-CGH technique using the Agilent platform. The SurePrint G3 Human CNV 2x400K Oligo Microarray kit (complete coverage of known CNVs) was used according to a modified protocol of Agilent's procedures. Briefly, genomic DNA was isolated and amplified, then purified using QIA-Miniprep_ Kit (QIAGEN) following manufacturer’s instructions and quantified using Nano_Drop Spectrophotometer. The DNA was labeled with Cy3-dUTP, the reference DNA, sex matched human genomic DNA was labeled with Cy5-dUTP. The labeled test and reference DNA were combined and purified, and then loaded onto the chips and hybridized according to manufacturer’s instructions. Images of the array were acquired with Agilent scanner G2505C (HD) and analyzed with Feature Extraction Software v3.0.5.1 (Agilent Technology, Santa Clara, CA, USA) under designed parameters: Genome: hg 18, Aberration Filters: min Probes = 3, DNA min Avg Abs Log Ratio = 0.25 and max Aberrations = 100,000 and per cent penetrance = 0. Finally Cyto Report file was created and the Cytogenomics software (Agilent Cytogenomics v3.0.6.6) was used for data analysis. Several online genetic databases were referred to during analysis of the results: the Database of Genomic Variants (DGVs), UCSC Genome browser on human Feb 2009 (GRCh 37/hg19 Assembly), and Database of Chromosomal Imbalance and Phenotype in Humans using Ensemble Resources (DECIPHER).

### Quantitative real time PCR (qPCR)

qPCR was performed on 25 samples (20 patients and 5 parents) using quantitative analysis and standard curve method [16]. We used SYBR Green Gene Expression Assays to detect the deletion on chromosome 7q11.23. The starting copy number of the unknown samples was determined using the comparative Ct method as previously described [19]. PCR primers’ sequences for amplification of microsatellite markers were selected from the USCS database sequences of chromosome 7 from position 71.449.000 to 73.925.000 as previously described [16]. Twelve PCR primer pairs are generating amplified fragments in 100–300 kb intervals along the WBS deletion region covering 2.5 Mb. The SOX9 gene on chromosome 17 or the B2M2 genes were used as reference gene (s) for confirmation of the array-CGH results. PCR was carried out using an ABI StepOne Plus (Applied Biosystems) in a 96-well optical plate with a final reaction volume of 10 μl. All primers were prepared at 100 pmol/μl and all DNA samples were diluted at 10 ng/μl. PCR master mix consisted of the appropriate volumes of KAPA SYBR® FAST qPCR Master Mix (2X), High ROX, primers, template DNA and RNase-grade water up to 10 μl. Thermal cycling conditions consisted of initial denaturation at 95 °C for 3 min, followed by 40 cycles of 95 °C for 15 s, 60 °C for 30 s and 72 °C for 1 min. The fractional cycle-number (Ct) of a probe, where the measured fluorescence reaches a fixed threshold is directly related to the amount of input DNA. Melting curve analysis was performed to identify the presence of primer-dimers and the specificity of the reaction. Analysis of the results was done by using StepOne™ Software V2.3 (Applied Biosystems). A higher or lower starting copy-number of input DNA as a sign for a deletion or a duplication will result in an earlier or later increase of fluorescence. Quantification of target sequence is normalized and relative copy number (RCN) determined on the basis of comparative ΔΔCt method with a normal control DNA as the calibrator. The ΔΔCt is calculated as follow: ΔΔCt = (ΔCt unknown sample-ΔCt control sample). Normalized copy number = 2^ˉΔΔCt^ A 0.5-fold RCN is used for deletion and 1.5-fold for duplication [19].

## Results

**Fig. 1:**
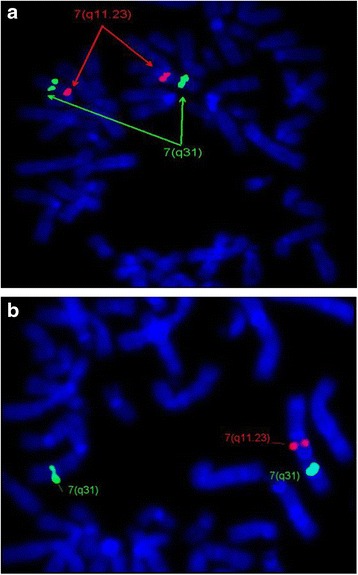
**a** FISH image shows no deletion in chr7q11.23. ELN probe showed two red signals on both chromosomes 7q11.23. **b** FISH image is showing the deletion of chr7q11.23. ELN probe showed only one red signal on one chromosome and absent signal on the other chromosome indicating deletion of 7q11.23
- Array-CGH technique has shown deletion in 8/22 patients (36 %), however, three patients and two parents have shown duplication in chromosome7q11.23 critical region for WBS. Five samples had approximate similar deletion sizes: sample (No.bl-680-11; bl-1787; and bl-858-14) have deletion size (1.42 Mb), samples bl-320 and bl-272 have deletion size (1.41 Mb), and (1.40 Mb) respectively. However, samples No. bl-1071-11; bl-232-15, and bl-402-15 had a larger deletion size (1,44 Mb) and (1.63 Mb) respectively. The size of deletion was confirmed by qPCR. Patients (bl-664-10) and bl-1190) did not have enough DNA for array-CGH analysis and were detected by qPCR only, their sizes were (1.8 Mb) and (1.40 Mb) respectively. Patient (680–11) has shown deletion in chromosome 7q11.23 (72359696–73780263) (Fig. 2) and duplication in chromosome 22q11.21 (17031614–19362298) (2330 Mb) (Fig. 3), inherited from her mother who had the same duplication variant at chromosome22q11.21 (17274835–19327233) (2.052 Mb). Patient (320–12) had dup22q13.1 (35944612–37522778) (1578 Mb) inherited from her mother who had dup 22q12.3-q13.1 (34759898–38422701) (3662 Mb) Other Copy Number Variants (CNVs) such as microdeletions or micro duplications were detected in other chromosomes, a summary of the CNVs observed in patients and their parents are listed in (Table 1). Figure 4 presents the sizes of deletions in chromosome 7q11.23 in all samples.

- Cytogenetic analysis using GTG-banding revealed no deletion in chromosome 7q11.23 in all samples, except one sample had translocation chromosomes 18;19 the karyotype was45,XX,der(19)t(18;19) (q11.1;p13.3)-18.
- FISH Analysis has shown the deletion on chromosome 7q11.23 in 10/24 (41.6 %) patients, but no deletion was observed in the parents. Example of a normal chromosome (7q) and deletion in chromosome 7q11.23 is shown in Fig. 1.

**Fig. 2:**
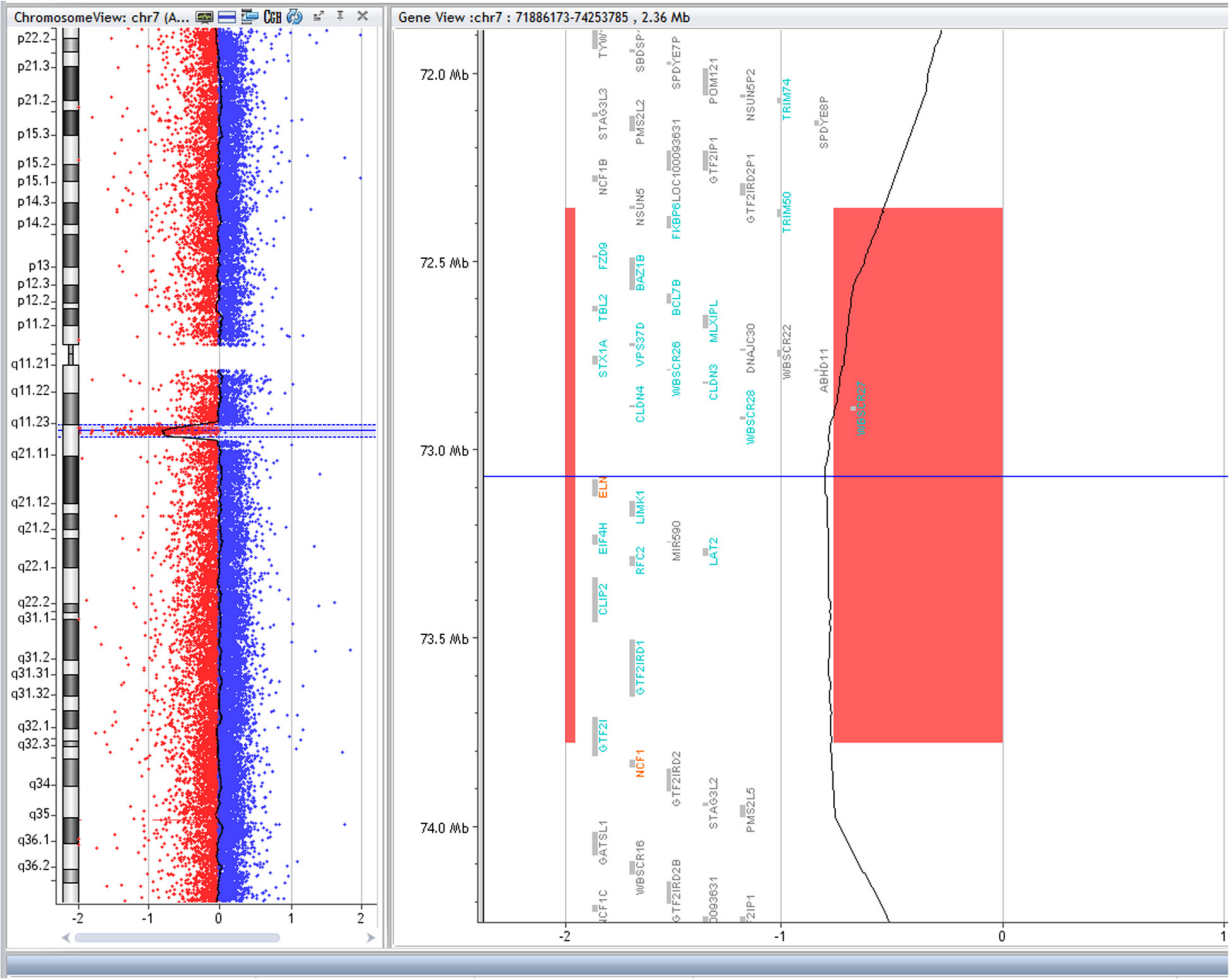
Array-CGH for chromosome7q (Agilent 2X400) showing deleted segment in chromosome 7q11.23 in patient (bl- 680–11), size of deletion 1.42 Mb, the deleted genes are shown within the deleted region

**Fig. 3:**
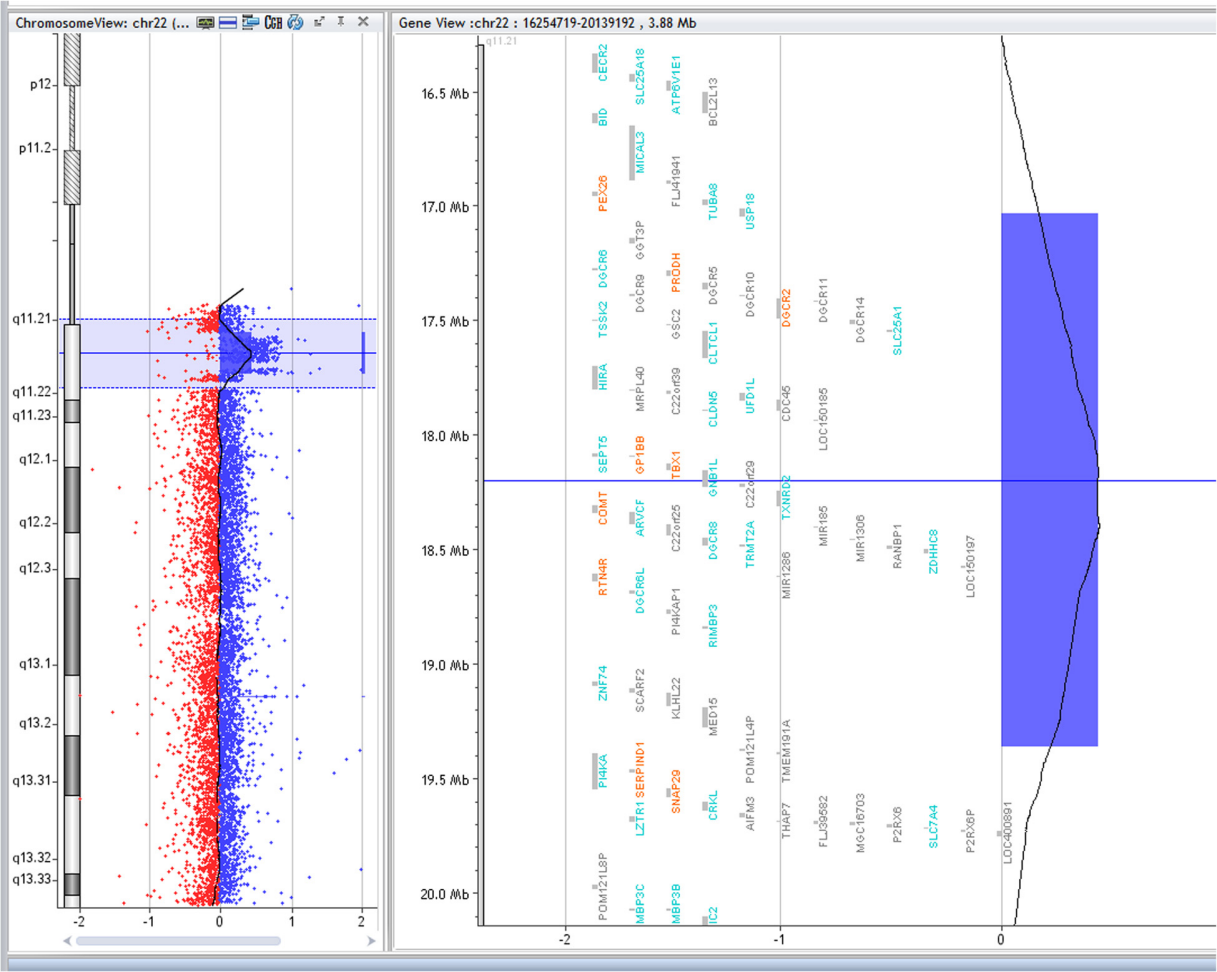
Array-CGH for chromosome 22 showing segmental duplication in the long arm of chromosome 22 (22q11.21) in case (bl-680-11) (inherited), the same duplication was found in both patient and her mother

**Table 1:** Summary of CNVs detected by array-CGH among Patients with Williams Syndrome

Sample	Relation	Del/ Dup cytoband	Start-stop (bp)	Del/dup size (bp)	
BL-680-11	WBS	Del 7q11.23	72359696–73780263	1,420,568	De Novo
		Dup 22q11.21 Del 10q11.21–q11.22 Del 15q11.2	17031614–19362298 45489823–46532996 18741716–20060120	2,330,685 dup 1,043,174 del 1,318,405	Maternal – Maternal
Bl–744–10	Mo. Of 680	Dup 22q11.21 Dup 15q11.2	17274835–19327233 18741716–20042737	2,052,398 1,301,022	–
BL-320–12	WBS	Del 7q11.23 Dup 7q11.23 Dup 16p11.2 Dup 22q13.1	72367665–73777326 75025883–75856615 27875036–30927847 35944612–37522778	1,409,662 830,733, 3,052,812 1,578,167	De novo Mat Mat. Mat.
Bl-1771–10	Mo of 320-12	Dup 7q11.23 Dup 16p11.2 Dup 22q12.3–q13.1	72635638–75982676 28110613–31427854 34759898–38422701	Dup 3.34 Mb 3,317,242 3,662,804	
BL-1810–10	CHD	Del 9p13.1–p11.2 Dup10q11.22 Del15q11.2	38758232–45359386 46388078–47970570 18818949–20308073	6,601,155 1,582,493 1,489,125	De novo
BL-1787–10	WBS	Del 7q11.23 Dup 10q11.22 Del 14q32.33 Del 15q11.2	72359696–73780263 46388078–47165895 105403001–15594248 18818949–19806019	1,420,568 777,818 191,248 987,071	De novo
Bl-272-14	WBS	Del 7q11.23	72382983–73780263	1,397	De novo
		Dup 16p13.3 Dup 11p15.4 Del 22q11.23	1961653–3066630 200300–2917590 22677959–22725353	1,105 Mb 2,717 Mb 47 Kb	–
BL-1071-11	WBS	Del 7q11.23 Dup 7q11.23 Dup 15q11.2	72338350–73780263 76271711–76421898 19537035–20366729	1,442 Mb 150 Kb 830 Kb	De Novo – Mat.
BL-402–15	WBS	Del 7q11.23 Dup 22q11.22	72730855–74339044 23056562–23245888	1.608 Mb 189 Kb	De Novo
Bl-1072-11	Mo. of 1071-11	Del 7q34 Dup 15q11.2	141413352–141438563 19465359–20418384	25 Kb 953 Kb	–
Bl–858-14	WBS Lt. Pulm. St	Del 7q11.23 Dup22q11.22	72721408–74139390 23056562–23245888	1,418 189 Kb	De Novo
BL-718-10	CHD	Dup 7q11.23	72635638–75885557	3,25 Mb	–
Bl-889-12	Dysmorphic Pulm .St	Dup7q11.22–q11.23	71073911–75851988	4,778 Mb	–
		Del 7q34 Del 15q11.2	141396899–141438563 18741716–20335946	41,665 Kb 1,594 Mb	–
BL-289-13	Pulm. St	Dup 7q11.22–q11.23	70573442–75877956	5,304,515	–

**Fig. 4:**
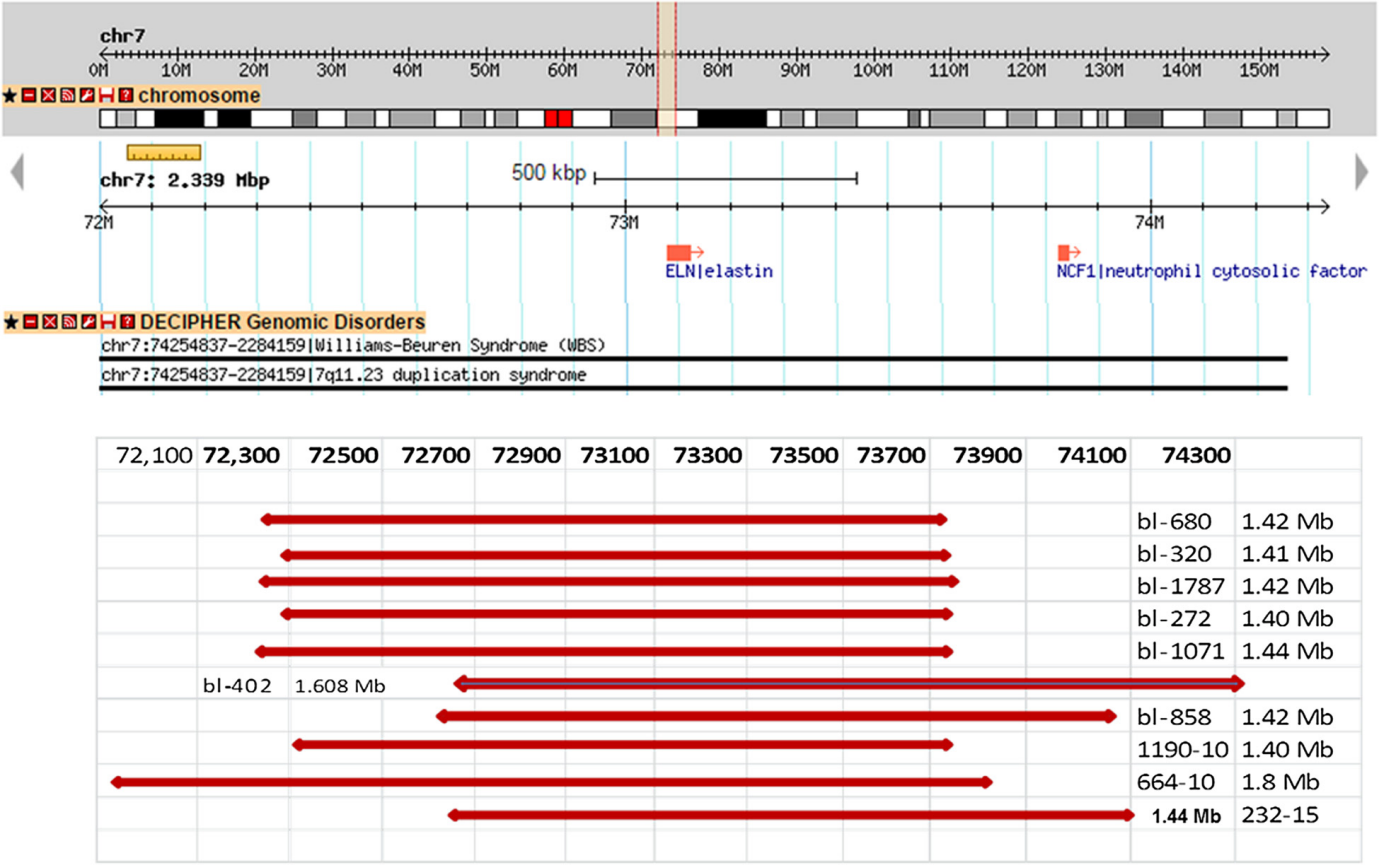
Map of chromosome 7q11.23 WBS critical region (http://www. Database of Genomic Variants) showing schematic representation of the deletions in ten patients. Six samples show typical deletion (1.40 - 1.44 Mb). Sample bl- 664-10 and bl-402 show larger deletions (1.8 and 1.6 Mb), samples bl-858 and bl-232 show different breakpoints but typical deletion size 1.42 and 1.44 Mb

**Fig. 5:**
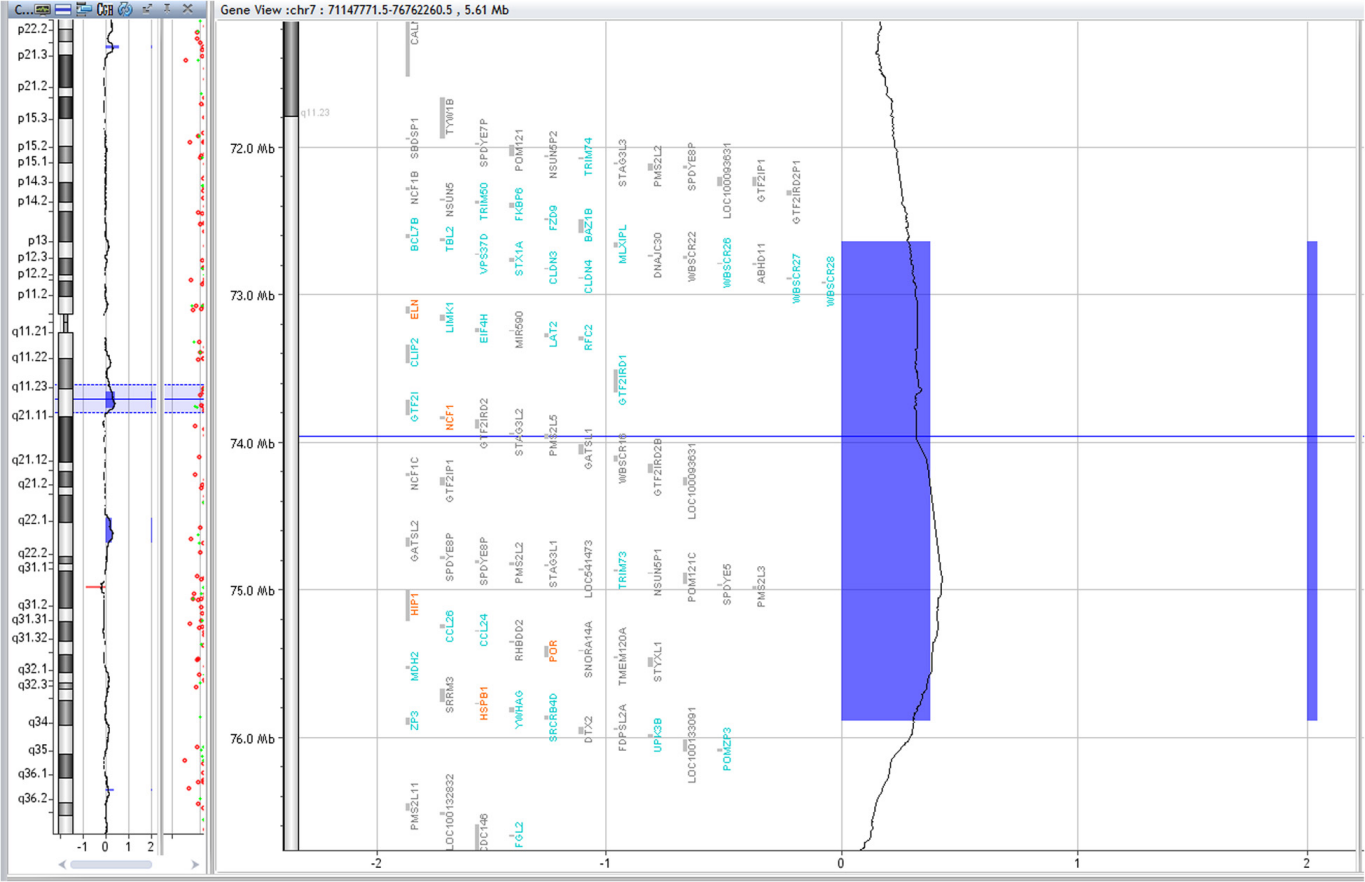
Chromosome 7 profile of the 2X400 array-CGH (Agilent) showing segmental duplication of chr 7q11.23 found in patient (bl-718-10), the genes included in the region (from 72635638 to 75885557) (3.249 Mb) are shown in the duplicated region

Three patients had no deletion by FISH analysis; however, array-CGH has shown duplication in chromosome 7q11.23. Patient (bl-718-10) had dup7q11.23 (72635638–75885557) (3.249 Mb) (Fig. 5), patient (bl-889-12) had dup 7q11.22-11.23 (71073911–75851988) (4.77 Mb), and patient (bl-289-13) had dup 7q11.23 (70573442–75877956) (5.304 Mb). The mother of case (bl-320-12) had duplication 7q11.23 (72635638–75982676) (3.347 Mb), and the mother of case bl-1071 has dup 7q11.23 (76,271,711-76,321,898) (150 kb).

Three patients not showing deletion at chromosome 7q WBS critical region had shown deletion in other chromosomes such as case (bl-663) has deletion chr18p11.32- p11.21 (14.859 Mb), case (bl-1127) has del 1p36.13, and case (bl-1810) has del 9 p13.1-p11.2 (38758232–45359386) (6601 Mb). Patient (bl-663) has translocation between chromosomes (18, 19) which could not be detected by array-CGH; the deletion in chr18p11 was reported differently by array-CGH and GTG-banding with (different breakpoints), because there are no probes at the centromeric region in array-CGH scanning (no probes at the position 18q11.1). Table 2 summarizes the clinical data, results of FISH analysis, array-CGH and qPCR techniques.

**Table 2:** Summary of the clinical data, results of FISH‚ array-CGH and qPCR analysis in patients with WBS

	Age/ Sex	Dysmorphic features	CHD	MR	Dental Anomalies	Hyper calcemia	FISH	Array-CGH del. size
680-11	8 yr/ F	+	SVAOS	+	+	No	Del 7 (q11.23)	Del 7q 1.42 Mb
Bl-744-10	35 yr/F	Mother of case No. 680-11	–	–	–	–	–	Dup 22q11.21
664-10	6 yr/M	+ features of WBS	SVAOS	+	+		Del 7 (q11.23)	Del 7q (qPCR) 1.8 Mb
1190-10	9 yr/F	SVAOS, Medullary nephrocalcinosis	SVAOS, AoCo, mild valvular Pul. st	+	+	No, high phosph	Del ch.7 (q11.23)	Del 7q (qPCR) 1.40 Mb
1071-11	18 yr/F	Characteristic features of WBS	SVAOS	+	+	yes	Del 7q (q11.23)	Del 7q 1.44 Mb
320-12	3 yr/F	Dysmorphic Mild LPA stenosis	SVAOS	+	+	No	Del 7q (q11.23)	Del 7q 1.41 Mb
1771-10	29 yr/ F	Mother of case No. 320-12	–	–	–	–	No del	Dup 7q11.23 3.347 Mb
1787-10	4 yr/F	+ Inguinal hernia	Congenital Pul. valve stenosis	+	+	No. High phosph	Del 7q (q11.23)	Del 7q 1.42 Mb
272-14	2 yr/M	+ Inguinal hernia, short stature	Pul. St, ASD, VSD	+	+		Del 7q (q11.23)	Del 7q 1.39 Mb
858-14	5y/ F	+ features of WBS	Pulm. St, PFO	+	+	+	Del 7q11.23	Del 7q 1.42 Mb
663-10	14 yr/F	Dysmorphic, speech delay, MR	Aortic stenosis	+	–	–	45,XX, der (19) t(18:19), (q11.1;p13.3	Del 18p11.32–p11.21 (14.859 Mb)
1140-11	7 yr/F	Squint, bulbous nose, flat nasal bridge, wide mouth, periorbital fullness, MR	VSD	+	+	No	Not done	Del 7q 0.2 Mb by qPCR
718-10	2 yr/F	dil. Cardiomyopath seizure, failure to thrive, left vocal cord paralysis	Trivial mitral regurge, trivial pulmonary. insufficiency	+	–	Cong. Hypo- calcemia	No del	Dup 7q11.23 (3.249 Mb)
889-12	1.1 yr/ F	dysmorphic	Supra valvular Ao. St., Lt pulmon.st		−	−	No del	Dup 7q11.23
289-13	1.5 yr F	DD, microcephaly, low set ears, epilepsy, semian crease	Pulm. St.	−	–	−	No del 7q	Dup7q11.22–q11.23 −

*Abbreviations*: *Del* deletion, *Chr* chromosome, *der* derivative, *q* long arm, *p* short arm, *F* female, *M* male, *yr* year, *ND* not done, *Pul. St*. pulmonary stenosis, *SVAOS* supra valvular Aortic stenosis

Five mothers and three fathers were available for array-CGH analysis, no deletion in chr7q was observed in all samples; however, qPCR has shown interrupted regions of deletions in one father and one mother which was not observed by array-CGH analysis.

### Q-PCR technique

**Fig. 6:**
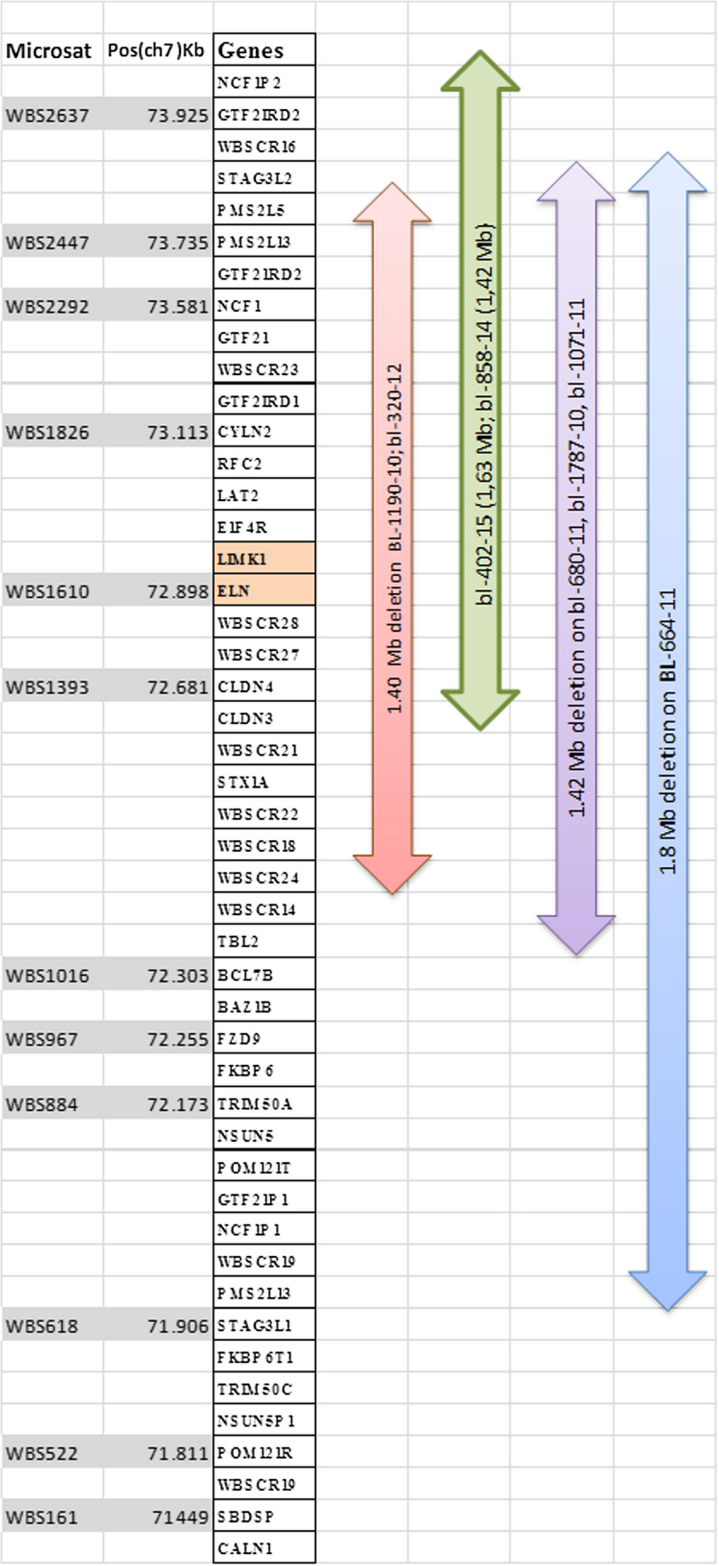
Graphic illustration of chromosome 7q11.23 showing the deleted regions of Williams-Beuren syndrome (with magnification of the region from nucleotides 71449–73.925) is showing the twelve microsatellite markers as grey bars that were assessed by qPCR. The vertical bars at the right side of the figure indicate the deleted regions of different sizes in our patients (adapted from Schubert and Laccone, 2006) [16]

The deletion in chromosome 7q11.23 was confirmed by qPCR: Six samples (sample No.Bl-680, bl-320, bl-1787, bl-1071, bl-272 and bl-1190) had common deletion sizes range between 1.40 and 1.44 Mb starting from markers WBS1016 to WBS2447. Larger deletion (1.61 Mb and 1.80 Mb) was identified in sample (bl-402-15 and Bl-664-10) starting from STS marker WBS2447 to WBS522. Figure 6 illustrates graphic representation of chromosome 7q11.23 presenting variable sizes of deletions found in the patients and the corresponding markers and their positions on the chromosome.

## Discussion

WBS is reliably detected by FISH technique, however, no deletions on chromosome 7q could be detected by conventional GTG banding [20]. Although visible deletion could be detected by high resolution technique [21] FISH technique is more sensitive to detect small changes on one chromosome. Specific probes using ELN and LIMKI genes, the most common genes involved in WBS are designed for detection of deletion on metaphase and interphase chromosomes to confirm the clinical diagnosis. Elcioglu [22] reported deletion in 12 out of 14 patients of WBS using FISH analysis, two patients did not have deletions for the ELN gene [22]. Tassabehji [23] used cosmids containing LIMK1, ELN and syntaxin 1A (STXIA). They detected deletion of LIMK1 in all subjects, while ELN was deleted in two patients but not the other subjects suggesting that the gene was either not deleted or only partially deleted. One drawback for FISH is that it cannot cover the whole deleted region on chromosome 7q11.23 [24] ranging in deleted lengths from patient to patient. Whereas qPCR proved to be useful for detection of the deletion size in WBS region with resolution of 100- to 300-Kb intervals [16]. Twelve pair of primers covering the deleted segment on 7q11.23 allowed for the detection of deletion size. This is probably due to the high sensitivity and the specificity of qPCR which can detect small copy number changes that cannot be detected by FISH. Deletion sizes in our patients ranged between 1.40 Mb and 1.44 Mb (80 %), while two samples had larger deletions (1.61 and 1.80 Mb). Schubert & Laccone [16] used qPCR to scan 2.5 Mb of the WBS deletion region at a resolution of 100-300Kb. They found that 21/65 (32 %) patients had deletion in the WBS region, (38 %) of patients had deletion size of (1.4 Mb) and (33 %) displayed (1.7 Mb) deletion and three others have a 1.8 Mb while one patient had a 200Kb deletion and another one has the large 2.5 Mb deletion [16].

Several studies reported that the most common deletion size is 1.55 Mb which is present in >95 % of individuals diagnosed as WBS due to mis-pairing between the B-centromeric and B-medial LCR blocks [25, 26]. Bayes et al. [12] reported two common deletion regions, 1.5 Mb deletion in 95 % of patients, while 5 % showed a larger deletion (1.8 Mb) due to abnormal recombination between block Ac and Am [12]. However, smaller deletion of about 850Kb was observed by Botta et al., [27] in two patients showing the full spectrum of WBS phenotype. Dutra et al., [28, 29] used microsatellite DNA markers and MLPA technique for detection of deletion size. They found the 1.55 Mb deletion in (90.5) and (89 %) of patients, and the 1.84 Mb deletion in (9.5) and (10.9 %) patients respectively.

Microarray—based CGH technique proved to be a reliable technique for dosage detection of genome variants. We could detect duplication of the WBS critical region in three patients and two mothers that could not be detected by FISH. The patients were referred to our Centre for the confirmation or/exclusion of WBS diagnosis. One patient (bl-718) (2 years’ old, female) presented with dysmorphic features, failure to thrive, congenital hypocalcemia, left vocal cord paralysis, seizures, delayed speech and dilated cardiomyopathy. The other patient (bl-889) had supravalvular aortic stenosis, left pulmonary stenosis and dysmorphic features. The third patient (bl-289-13) presented with developmental delay, speech delay, microcephaly, low set ears, epilepsy and pulmonary stenosis. It is interesting to note that the first case of Dup7q11.23 was accidentally found in a child referred for evaluation of velocardiofacial syndrome [30]. Torniero et al. [31] reported a patient with duplication reciprocal to the WBS critical region on chr7q11.23 with dysmorphic features, speech delay, pachygyria and epilepsy, and proposed that at least one gene in the WBS critical region can impair neuronal migration. Beunders et al. [32] described the reciprocal duplication in 27 families, and triplication in another patient with speech delay, behavioral problems, dysmorphism, and suggested that amplification of the WBS region is disease causing with incomplete penetrance. More recent reports documented 7q11.23 duplication syndrome as a genetic disorder associated with speech/language delay, characteristic features, hypotonia and developmental delay [33, 34]. Our results confirmed that duplication of 7q WBS critical region is disease causing and patients are mainly presented with dysmorphic features, speech delay, intellectual disability, and congenital heart defects.

We observed dup7q11.23 in the mother of case (bl-320), the presence of duplication on chromosome 7q11.23 in the mother of a case with chr 7q11.23 deletion was previously observed by Torniero [31]. They reported the presence of duplication (7q11.23) in parents of probands with deletion or duplication in WBS critical region. The parents did not have any speech or cognitive impairment. Structural variants can increase the risk of secondary rearrangements causing disease in the offspring, Other studies [26, 35] suggested that segmental duplication (Dup 7q11.23) or inversion in the WBS critical region (WBS inv-1 variant) confer an increased risk factor for WBS deletion mediated by misalignment and non-allelic homologous recombination [26].

The impact of the deleted genes on the phenotype can be explained from other studies of gene functions. The most common deletion size (1.40 Mb-1.55 Mb) involved approximately 25–28 genes. The mostly described morbid genes were ELN, LIMK1. Elastin (ELN) gene (MIM 130160) was described to have a role in arterial development; it was shown that interruption of ELN expression leads to profound arterial thickening and increased risk of obstructive vascular disease [36], furthermore, ELN gene mutation is causative for isolated supravalvular aortic stenosis (SVAS), and can cause autosomal dominant SVAS [37, 38]. LIMK1 gene is expressed in the central nervous system during embryogenesis, including the inner nuclear layer of the retina, the cortex, the developing spinal cord, and dorsal root ganglia; it was identified as a strong candidate for the neurologic features of WBS [39].

Other commonly deleted genes are the WBSCR22 gene; its protein is predicted to have methyltransferase activity. Hemizygous deletion of this gene might contribute to the growth retardation, the myopathy or the premature aging effects in the pathogenesis of WBS [40]. The CLDN3 and CLDN4 belong to the CLAUDINS family; they are components of epithelial cell tight junctions and may play a role in internal organ development and function during pre- and postnatal life.

Variability in the deletion size may affect different number of genes, larger deletion was observed in two patients patient bl-402 has a deletion (1.63 Mb) involving (31) genes, the extra genes are (NCF1, GTF2IRD2, STAG3L2, PMS2P5) genes. The NCF1 gene, a NAD(P)H oxidase subunit, was shown to be involved in the generation of oxidative stress and can modify vascular stiffness observed in WBS patients [41]. Further studies on mouse model have linked haplo-insufficiency including elastin gene to increased vascular stiffness and hypertension [42]. It was observed that extension of the WBS deletion size to involve NCF1 gene was associated with protection from vascular stiffness and hypertension in WBS patients [41, 43]. This observation is useful for management of WBS patients by using anti- hypertensive and agents inhibiting oxidative stress for the protection from these illnesses [43]. Dutra et al. [28] indicated that SVAS as well as ocular and urinary abnormalities were more common in patients with a large deletion. Several studies reported no relationship between the size of the deletion and clinical features [28, 44, 45], and no difference in the frequency of maternal or paternal deletions, or the effect of the origin of deletion on the phenotype [28]. However, others suggested a parent of origin effect on microcephaly and growth retardation [46, 47]. Deletions in all of our samples are de novo; therefore no effect of parent of origin could be implicated in this study.

We observed that two patients who had no deletion by FISH or array-CGH, showed deletion of sporadic regions on chromosome 7q11.23 using qPCR. It was suggested that partial microdeletion can lead to false negative result by FISH probes. [27, 28, 48]. These patients do not have typical features of WBS and could be caused by deletion/or mutations in other genes which require further analysis.

Two patients (bl-680-11) and (bl-320) had duplication in chromosome 22q11.2 that was inherited from maternal side, the two mothers were apparently normal. Previous reports confirmed that micro duplication of 22q11.2 is frequently inherited and the majority of parents with 22q11.2 duplication showed no or minor abnormalities [49, 50]. It was observed that chromosome 22q11.2 contains multiple region-specific low copy repeats (LCR) which mediate genomic rearrangement during meiosis resulting into variable phenotypes [49], however, the size of the inherited duplication might be slightly different (e.g. case 680 and her mother). This could be a technical limitation in the array-CGH scanning, (the CNV calling algorithm is calling the ends of the duplication differently between the two samples due to probe density or type of probe used and the actual breakpoints are the same) [51].

Previous studies indicated the difficulty in clinical diagnosis of some patients presenting with overlapping features with other syndromes and not having typical heart defects characteristic of WBS and highlighted the importance of microarray analysis in the clinical diagnosis of these complex genetic conditions [52]. Three patients in our study were found to have deletion in other chromosomes: 1p36.13, 18p11.32p11.21, and 9p13.1-p11.2. Congenital heart defects associated with DD, mental retardation and other dysmorphic features were reported in 1p36 deletion syndrome [53], and partial monosomy18p [54], however, no cardiac defects were reported in deletion 9p11.2-p13.1.

## Conclusions

We can conclude that FISH and array-CGH are reliable and efficient methods for diagnosis of WBS. However, Array-CGH has the advantage of scanning the whole genome for regions of duplication /or deletion in cases with syndromic CHDs not clearly identified as WBS, allowing for the early management and better prognosis of affected cases. Furthermore, both array-CGH and qPCR are useful for detection of the break points on the chromosome and can identify the genes that are interrupted on the deleted region and their impact on the disease phenotype.

## Abbreviations

Array-CGH, array-comparative genomic hybridization; CHDs, congenital heart defects; WBS, Williams –Beuren syndrome; WS, Williams’ syndrome; FISH, Fluorescent in-Situ Hybridization; qPCR, quantitative real-time polymerase chain reaction; LCR, low copy repeat; CEGMR, Center of Excellence in Genomic Medicine Research; KAU, King AbdulAziz University; KAUH, King Abdulaziz University Hospital; DGMU, Diagnostic Genomic Medicine Unit; DGVs, Database of Genomic Variants; DECIPHER, Database of Chromosomal Imbalance and Phenotype in Humans using Ensemble Resources; PCR, polymerase chain reaction; RCN, relative copy number; CNVs, copy number variants; SA, Saudi Arabia; SVAS, supravalvular aortic stenosis

## References

[1] Alabdulgader AA (2001). Congenital heart disease in 740 subjects: epidemiological aspects. Ann Trop Paediatr.

[2] Fida NM, Al-Aama J, Nichols W, Nichols W, Alqahtani M (2007). A prospective study of congenital malformations among live born neonates at a University Hospital in Western Saudi Arabia. Saudi Med J.

[3] Greer W, Sandridge AL, Al-Menieir M, Al Rowais A (2005). Geographical distribution of congenital heart defects in Saudi Arabia. Ann Saudi Med.

[4] Alqurashi M, Mouzan ME, Herbish AA, Salloum AA, Omer[Double Dagger] AA (2007). Symptomatic congenital heart disease in the Saudi children and adolescents project. Ann Saudi Med.

[5] Williams JC, Barratt-Boyes BG, Lowe JB (1961). Supravalvular aortic stenosis. Circulation.

[6] Beuren AJ, Apitz J, Harmjanz D (1962). Supravalvular aortic stenosis in association with mental retardation and a certain facial appearance. Circulation.

[7] Preus M (1984). The Williams syndrome: objective definition and diagnosis. Clin Genet.

[8] Martin ND, Snodgrass GJ, Cohen RD (1984). Idiopathic infantile hypercalcaemia--a continuing enigma. Arch Dis Child.

[9] Morris CA, Thomas IT, Greenberg F (1993). Williams syndrome: autosomal dominant inheritance. Am J Med Genet.

[10] Stromme P, Bjornstad PG, Ramstad K (2002). Prevalence estimation of Williams syndrome. J Child Neurol.

[11] Shaffer LG, Lupski JR (2000). Molecular mechanisms for constitutional chromosomal rearrangements in humans. Annu Rev Genet.

[12] Bayes M, Magano LF, Rivera N, Flores R, Perez Jurado LA (2003). Mutational mechanisms of Williams-Beuren syndrome deletions. Am J Hum Genet.

[13] Valero MC, de Luis O, Cruces J, Perez Jurado LA (2000). Fine-scale comparative mapping of the human 7q11.23 region and the orthologous region on mouse chromosome 5G: the low-copy repeats that flank the Williams-Beuren syndrome deletion arose at breakpoint sites of an evolutionary inversion(s). Genomics.

[14] Karmiloff-Smith A, Grant J, Ewing S, Carette MJ, Metcalfe K, Donnai D, Read AP, Tassabehji M. Using case study comparisons to explore genotype-phenotype correlations in Williams-Beuren syndrome. J Med Genet. 2003;40(2):136–40. 10.1136/jmg.40.2.136 PMC1735363 12566524

[15] Tassabehji M (2003). Williams-Beuren syndrome: a challenge for genotype-phenotype correlations. Hum Mol Genet.

[16] Schubert C, Laccone F (2006). Williams-Beuren syndrome: determination of deletion size using quantitative real-time PCR. Int J Mol Med.

[17] Rothlisberger B, Hoigne I, Huber AR, Brunschwiler W, Capone Mori A (2010). Deletion of 7q11.21-q11.23 and infantile spasms without deletion of MAGI2. Am J Med Genet A.

[18] Antonell A, Del Campo M, Magano LF, Kaufmann L, de la Iglesia JM, Gallastegui F, Flores R, Schweigmann U, Fauth C, Kotzot D, et al. Partial 7q11.23 deletions further implicate GTF2I and GTF2IRD1 as the main genes responsible for the Williams-Beuren syndrome neurocognitive profile. J Med Genet. 2010;47(5):312–20. 10.1136/jmg.2009.071712 19897463

[19] Vanguilder HD, Vrana KE, Freeman WM (2008). Twenty-five years of quantitative PCR for gene expression analysis. Biotechniques.

[20] Lowery MC, Morris CA, Ewart A, Brothman LJ, Zhu XL, Leonard CO, Carey JC, Keating M, Brothman AR. Strong correlation of elastin deletions, detected by FISH, with Williams syndrome: evaluation of 235 patients. Am J Hum Genet. 1995;57(1):49–53. PMC1801249 7611295

[21] Wu YQ, Nickerson E, Shaffer LG, Keppler-Noreuil K, Muilenburg A (1999). A case of Williams syndrome with a large, visible cytogenetic deletion. J Med Genet.

[22] Elcioglu N, Mackie-Ogilvie C, Daker M, Berry AC (1998). FISH analysis in patients with clinical diagnosis of Williams syndrome. Acta Paediatr.

[23] Tassabehji M, Metcalfe K, Karmiloff-Smith A, Carette MJ, Grant J, Dennis N, Reardon W, Splitt M, Read AP, Donnai D. Williams syndrome: use of chromosomal microdeletions as a tool to dissect cognitive and physical phenotypes. Am J Hum Genet. 1999;64(1):118–25. 10.1086/302214 PMC1377709 9915950

[24] Nickerson E, Greenberg F, Keating MT, McCaskill C, Shaffer LG (1995). Deletions of the elastin gene at 7q11.23 occur in approximately 90 % of patients with Williams syndrome. Am J Hum Genet.

[25] Meng X, Lu X, Li Z, Green ED, Massa H, Trask BJ, Morris CA, Keating MT. Complete physical map of the common deletion region in Williams syndrome and identification and characterization of three novel genes. Hum Genet. 1998;103(5):590–9. 10.1007/s004390050874 9860302

[26] Merla G, Brunetti-Pierri N, Micale L, Fusco C (2010). Copy number variants at Williams-Beuren syndrome 7q11.23 region. Hum Genet.

[27] Botta A, Novelli G, Mari A, Novelli A, Sabani M, Korenberg J, Osborne LR, Digilio MC, Giannotti A, Dallapiccola B. Detection of an atypical 7q11.23 deletion in Williams syndrome patients which does not include the STX1A and FZD3 genes. J Med Genet. 1999;36(6):478–80. PMC1734394 10874638

[28] Dutra RL, Pieri Pde C, Teixeira AC, Honjo RS, Bertola DR, Kim CA (2011). Detection of deletions at 7q11.23 in Williams-Beuren syndrome by polymorphic markers. Clinics (Sao Paulo).

[29] Dutra RL, Honjo RS, Kulikowski LD, Fonseca FM, Pieri PC, Jehee FS, Bertola DR, Kim CA. Copy number variation in Williams-Beuren syndrome: suitable diagnostic strategy for developing countries. BMC Res Notes. 2012;5:13. 10.1186/1756-0500-5-13 PMC3285034 22226172

[30] Somerville MJ, Mervis CB, Young EJ, Seo EJ, del Campo M, Bamforth S, Peregrine E, Loo W, Lilley M, Perez-Jurado LA, et al. Severe expressive-language delay related to duplication of the Williams-Beuren locus. N Engl J Med. 2005;353(16):1694–701. 10.1056/NEJMoa051962 PMC2893213 16236740

[31] Torniero C, Dalla Bernardina B, Novara F, Cerini R, Bonaglia C, Pramparo T, Ciccone R, Guerrini R, Zuffardi O. Dysmorphic features, simplified gyral pattern and 7q11.23 duplication reciprocal to the Williams-Beuren deletion. Eur J Hum Genet. 2008;16(8):880–7. 10.1038/ejhg.2008.42 18337728

[32] Beunders G, van de Kamp JM, Veenhoven RH, van Hagen JM, Nieuwint AW, Sistermans EA (2010). A triplication of the Williams-Beuren syndrome region in a patient with mental retardation, a severe expressive language delay, behavioural problems and dysmorphisms. J Med Genet.

[33] Velleman SL, Mervis CB (2011). Children with 7q11.23 Duplication Syndrome: Speech, Language, Cognitive, and Behavioral Characteristics and their Implications for Intervention. Perspect Lang Learn Educ.

[34] Mohan S, Nampoothiri S, Yesodharan D, Venkatesan V, Koshy T, Paul SF, Perumal V. Reciprocal Microduplication of the Williams-Beuren Syndrome Chromosome Region in a 9-Year-Old Omani Boy. Lab Med. 2016;47(2):171–5. 10.1093/labmed/lmw005 27069036

[35] Cusco I, Corominas R, Bayes M, Flores R, Rivera-Brugues N, Campuzano V, Perez-Jurado LA. Copy number variation at the 7q11.23 segmental duplications is a susceptibility factor for the Williams-Beuren syndrome deletion. Genome Res. 2008;18(5):683–94. 10.1101/gr.073197.107 PMC2336808 18292220

[36] Li DY, Brooke B, Davis EC, Mecham RP, Sorensen LK, Boak BB, Eichwald E, Keating MT. Elastin is an essential determinant of arterial morphogenesis. Nature. 1998;393(6682):276–80. 10.1038/30522 9607766

[37] Li DY, Toland AE, Boak BB, Atkinson DL, Ensing GJ, Morris CA, Keating MT. Elastin point mutations cause an obstructive vascular disease, supravalvular aortic stenosis. Hum Mol Genet. 1997;6(7):1021–8. 10.1093/hmg/6.7.1021 9215670

[38] Park S, Seo EJ, Yoo HW, Kim Y (2006). Novel mutations in the human elastin gene (ELN) causing isolated supravalvular aortic stenosis. Int J Mol Med.

[39] Tassabehji M, Metcalfe K, Fergusson WD, Carette MJ, Dore JK, Donnai D, Read AP, Proschel C, Gutowski NJ, Mao X, et al. LIM-kinase deleted in Williams syndrome. Nat Genet. 1996;13(3):272–3. 10.1038/ng0796-272 8673124

[40] Doll A, Grzeschik KH (2001). Characterization of two novel genes, WBSCR20 and WBSCR22, deleted in Williams-Beuren syndrome. Cytogenet Cell Genet.

[41] Del Campo M, Antonell A, Magano LF, Munoz FJ, Flores R, Bayes M, Perez Jurado LA. Hemizygosity at the NCF1 gene in patients with Williams-Beuren syndrome decreases their risk of hypertension. Am J Hum Genet. 2006;78(4):533–42. 10.1086/501073 PMC1424678 16532385

[42] Campuzano V, Segura-Puimedon M, Terrado V, Sanchez-Rodriguez C, Coustets M, Menacho-Marquez M, Nevado J, Bustelo XR, Francke U, Perez-Jurado LA. Reduction of NADPH-oxidase activity ameliorates the cardiovascular phenotype in a mouse model of Williams-Beuren Syndrome. PLoS Genet. 2012;8(2), e1002458. 10.1371/journal.pgen.1002458 PMC3271062 22319452

[43] Kozel BA, Danback JR, Waxler JL, Knutsen RH, de Las Fuentes L, Reusz GS, Kis E, Bhatt AB, Pober BR. Williams syndrome predisposes to vascular stiffness modified by antihypertensive use and copy number changes in NCF1. Hypertension. 2014;63(1):74–9. 10.1161/HYPERTENSIONAHA.113.02087 PMC3932371 24126171

[44] Wang MS, Schinzel A, Kotzot D, Balmer D, Casey R, Chodirker BN, Gyftodimou J, Petersen MB, Lopez-Rangel E, Robinson WP. Molecular and clinical correlation study of Williams-Beuren syndrome: No evidence of molecular factors in the deletion region or imprinting affecting clinical outcome. Am J Med Genet. 1999;86(1):34–43. 10440826

[45] Pober BR (2010). Williams-Beuren syndrome. N Engl J Med.

[46] Perez Jurado LA, Peoples R, Kaplan P, Hamel BC, Francke U (1996). Molecular definition of the chromosome 7 deletion in Williams syndrome and parent-of-origin effects on growth. Am J Hum Genet.

[47] Brondum-Nielsen K, Beck B, Gyftodimou J, Horlyk H, Liljenberg U, Petersen MB, Pedersen W, Petersen MB, Sand A, Skovby F, et al. Investigation of deletions at 7q11.23 in 44 patients referred for Williams-Beuren syndrome, using FISH and four DNA polymorphisms. Hum Genet. 1997;99(1):56–61. 10.1007/s004390050311 9003495

[48] Heller R, Rauch A, Luttgen S, Schroder B, Winterpacht A (2003). Partial deletion of the critical 1.5 Mb interval in Williams-Beuren syndrome. J Med Genet.

[49] Ou Z, Berg JS, Yonath H, Enciso VB, Miller DT, Picker J, Lenzi T, Keegan CE, Sutton VR, Belmont J, et al. Microduplications of 22q11.2 are frequently inherited and are associated with variable phenotypes. Genet Med. 2008;10(4):267–77. 10.1097/GIM.0b013e31816b64c2 18414210

[50] Wentzel C, Fernstrom M, Ohrner Y, Anneren G, Thuresson AC (2008). Clinical variability of the 22q11.2 duplication syndrome. Eur J Med Genet.

[51] Scherer SW, Lee C, Birney E, Altshuler DM, Eichler EE, Carter NP, Hurles ME, Feuk L. Challenges and standards in integrating surveys of structural variation. Nat Genet. 2007;39(7 Suppl):S7–S15. 10.1038/ng2093 PMC2698291 17597783

[52] Honeywell C, Gardin L, Jimenez-Rivera C, Allanson J (2009). Array CGH ends diagnostic odyssey for infant with features of Williams and Alagille syndrome. Am J Med Genet A.

[53] Battaglia A, Hoyme HE, Dallapiccola B, Zackai E, Hudgins L, McDonald-Mcginn D, Bahi-Buisson N, Romano C, Williams CA, Brailey LL, et al. Further delineation of deletion 1p36 syndrome in 60 patients: a recognizable phenotype and common cause of developmental delay and mental retardation. Pediatrics. 2008;121(2):404–10. 10.1542/peds.2007-0929 18245432

[54] Schmidt B, Udink Ten Cate F, Weiss M, Koehler U (2012). Cardiac malformation of partial trisomy 7p/monosomy 18p and partial trisomy 18p/monosomy 7p in siblings as a result of reciprocal unbalanced malsegregation—and review of the literature. Eur J Pediatr.

